# Simultaneous bioremediation of cationic copper ions and anionic methyl orange azo dye by brown marine alga *Fucus vesiculosus*

**DOI:** 10.1038/s41598-021-82827-8

**Published:** 2021-02-11

**Authors:** Noura El-Ahmady El-Naggar, Ragaa A. Hamouda, Amna A. Saddiq, Monagi H. Alkinani

**Affiliations:** 1 Department of Bioprocess Development, Genetic Engineering and Biotechnology Research Institute, City of Scientific Research and Technological Applications (SRTA-City), New Borg El-Arab City, 21934 Alexandria Egypt; 2 Department of Biology, College of Sciences and Arts Khulais, University of Jeddah, Jeddah, Saudi Arabia; 3 Microbial Biotechnology Department, Genetic Engineering and Biotechnology, Research Institute, University of Sadat City, El Sadat City, Egypt; 4 Department of Biology, College of Sciences, University of Jeddah, Jeddah, Saudi Arabia; 5 Department of Computer Science and Artificial Intelligence College of Computer Science and Engineering, University of Jeddah, Jeddah, Saudi Arabia

**Keywords:** Environmental biotechnology, Environmental sciences, Applied microbiology, Environmental microbiology

## Abstract

Textile wastewater contains large quantities of azo dyes mixed with various contaminants especially heavy metal ions. The discharge of effluents containing methyl orange (MO) dye and Cu^2+^ ions into water is harmful because they have severe toxic effects to humans and the aquatic ecosystem. The dried algal biomass was used as a sustainable, cost-effective and eco-friendly for the treatment of the textile wastewater. Box–Behnken design (BBD) was used to identify the most significant factors for achieving maximum biosorption of Cu^2+^ and MO from aqueous solutions using marine alga *Fucus vesiculosus* biomass. The experimental results indicated that 3 g/L of *F. vesiculosus* biomass was capable of removing 92.76% of copper and 50.27% of MO simultaneously from aqueous solution using MO (60 mg/L), copper (200 mg/L) at pH 7 within 60 min with agitation at 200 rpm. The dry biomass was also investigated using SEM, EDS, and FTIR before and after MO and copper biosorption. FTIR, EDS and SEM analyses revealed obvious changes in the characteristics of the algal biomass as a result of the biosorption process. The dry biomass of *F. vesiculosus* can eliminate MO and copper ions from aquatic effluents in a feasible and efficient method.

## Introduction

Azo dyes and heavy metals are common pollutants worldwide^1^. Several industries are responsible for azo dyes and heavy metals pollutants, including leather tanning, pesticides, fuel, energy production, mining, atomic energy and batteries, surface finishing, textile, electroplating, metal plating, and photography^2^. If the effluents of these industries are not treated; it can cause environmental pollution and be dangerous to humans^2^.

Copper is essential to life because it plays catalytic and structural roles in some biomolecules such as proteins, enzyme, hemoglobin formation, carbohydrate metabolism and hair keratin^3^. Copper acts as a co-factor for several enzymes and plays a significant role in protein transcription^4^. Copper compounds are toxic through inhalation, eating, drinking, and dermal exposure to environmental conditions that contain copper^5^. In humans, toxicity of Cu is associated with two severe diseases, Menkes disease and Wilson’s disease, leading to the destruction of vital organs and alteration in lipid peroxidation in liver mitochondria and reduced liver and blood concentrations of the antioxidant vitamin E^6^. Chronic Cu toxicity initiate oxidative damage, neurodegenerative diseases as Alzheimer's, Parkinson’ and Huntington’s diseases, hepatic disorder (liver cirrhosis) and other disease conditions including Cu-protein interaction in the nerve system, temporal and spatial distribution of Cu in hepatocytes, activation of acidic sphingomyelinase and release of ceramide, alpha-synuclein aggregation, as well as lipid metabolism, genetic abnormality of Cu metabolism (Wilson’s disease) ^6^.

Azo dyes pollutants are generally highly coloured, and the immediate release of the azo dye pollutant into the water body causes the risk to both human and marine life as a result of their toxicity, mutagenicity and carcinogenicity impacts^7^. The quantity of azo dyes liberate to the environment is up 50% depending on the dye type and spread faster in the environments^8^. Methyl orange is a stable, water-soluble with less biodegrading ability. Hence, it is hard to eliminate MO from aqueous solutions by traditional treatment procedures^9^. The discharge of methyl orange into the aquatic environments causes major environmental problems, critical health issues, as well as a severe and long-term impact on marine life^10^. Methyl orange causes toxicity if it swallowed, inhaled, or contact to the dermis^11^.

Several physico-chemical approaches have been widely used for the elimination of metallic ions and azo dyes from the contaminated water including chemical sedimentation, complexion, foam flotation, coagulation, ion exchange, cementation, membrane operations solvent extraction and electro-deposition^12^. However, these conventional methods for dyes and heavy metals removal have been restricted due to high reagents and energy requirements, production of high amounts of toxic sludge, highly expensive, and also generation of other toxic substances^13^.

Therefore, there is an urgent need for alternative method to the physicochemical conventional methods for heavy metals and azo dyes removal. Consequently, bioremediation (biosorption) is a promising, simple, cheap, eco-friendly and efficient alternative method to the physicochemical conventional methods for treatment of contaminated wastewater^14,15^. The biosorption process had been used to remove toxic metal ions and hazardous azo dyes from hazardous effluents using natural biosorbents at low costs. Hamouda et al*.*^16^ reported that the application of biological materials such as the biomass of microorganisms including algae, bacteria, fungi and yeasts were used as biosorbents for wastewater pollutants. The marine algae biomasses are considered to be effective sustainable biosorbents with high ability for biosorption of pollutants such as hazardous azo dyes and heavy metals ions^17^ due to their the cell wall constitutes, its high metal-binding affinity, low-cost and local abundance^14,18^. Dried biomass of marine algae have been shown to be a promising, renewable biomass that are efficient biosorbents with a high binding efficiency to various pollutants in aqueous effluents including metals and dyes^14,18^. The cell wall of algae contains heteropolysaccharides, lipids and proteins which consist of various active sites (functional groups) that include carboxyl, amino, hydroxyl and phosphate groups that can act as cell surface binding sites to a variety of pollutants and binds with azo dyes or heavy metals ions through various mechanisms including surface precipitation, chelation, ion exchange, electrostatic forces, complexation between pollutants cations and the surface of the seaweeds or diffusion interior the cells, bioaccumulation inside the cells and binding to proteins and other intracellular components^16,19^.

Biosorption of the pollutants from aqueous solutions by biomass of brown algae has mainly been attributed to the highest alginate contents of their cell walls ^20^. Ahmady-Asbchin and Mohammadi^21^ reported that the highest bioremoval of copper from wastewater was obtained by the dried biomass of brown marine alga especially *Fucus vesiculosus* and *Fucus serratus*.

The Box–Behnken design (BBD)^22^ is a statistical experimental design used to determine the optimum conditions to achieve the maximum simultaneous removal of copper ions and methyl orange by *Fucus vesiculosus* biomass. Furthermore, the linear, quadratic and mutual interaction impacts among the selected variables of the biosorption process should be studied. Several researchers have applied Box–Behnken design for optimization of different independent variables^23,24^.

Numerous studies have been conducted on mono-pollutant biosorption, despite the fact that both dyes and heavy metal ions are present together in significant amounts in the industrial effluents. Therefore, this study was paid high priority to perform the simultaneous removal of copper ions and MO dye from the binary solution using the dry biomass of marine alga, *Fucus vesiculosus*, as a cost effective biosorbent, to optimize the factors affecting the biosorption process using Box-Behnken experimental design and to characterize the biomass of brown alga, *Fucus vesiculosus,* before and after the biosorption process.

## Results and discussion

The copper biosorption process by algae is strongly influenced by multiple factors such as initial pH, incubation time, and concentration of the copper^25^. On the other hand, decolorization of azo dye through biosorption processes is influenced by several factors including pH, algal biomass, incubation time and initial dye concentration^26,27^.

### Statistical optimization of simultaneous biosorption of copper and methyl orange by *Fucus vesiculosus* biomass

In the current study, the dried biomass of brown alga, *Fucus vesiculosus*, was used as biosorbent for simultaneous biosorption of MO and copper ions from aqueous solutions. Optimization of biosorption process independent variables was carried out using Box–Benken design to study the individual, quadratic and interaction effects between the different independent process variables and to maximize the removal percentages. Box–Benken design was also used to predict the best biosorption process conditions for maximum simultaneous biosorption of MO and copper ions from aqueous solutions. In the current experiment, the Box-Benken design of 29 experimental trials was used for optimization of the selected variables including MO conc. (X_1_), algal biomass (X_2_), initial pH level (X_3_) and incubation time (X_4_) on the biosorption of MO and copper ions from aqueous binary solutions.

Table 1 presents the levels of coded and actual values of the 4 independent factors. Additionally, experimental and theoretical predicted percentages of methyl orange and copper bioremoval along with the residuals are also presented in Table 1. According to the obtained experimental results of Box-Benken design, the percentages of MO removal are ranged from 0.31 to 50.27% and the copper ions removal percentages are ranged from 51.46 to 92.76%. The low removal percentages of MO (ranged from 0.31 to 50.27%) could be explained by the simultaneous biosorption of copper ions and MO dye by *Fucus vesiculosus* biomass. Consequently, due to high copper concentration used in this study (200 mg/L), competition between copper ions and MO for binding sites decreases the MO biosorption.

**Table 1:** Box–Behnken design matrix of four process variables with actual factor levels, coded factor levels, predicted and experimental values of simultaneous biosorption of methyl orange dye and copper (II) ions by using *Fucus vesiculosus.*

Std	Run	X_1_	X_2_	X_3_	X_4_	Methyl orange removal (%)			Copper removal (%)		
						Actual	Predicted	Residuals	Actual	Predicted	Residuals
10	1	1	0	0	− 1	7.30	6.72	0.58	77.77	77.94	− 0.17
14	2	0	1	− 1	0	20.30	19.34	0.96	57.52	57.96	− 0.44
1	3	− 1	− 1	0	0	30.69	29.43	1.26	57.79	57.73	0.06
12	4	1	0	0	1	39.30	40.33	− 1.03	73.33	73.13	0.20
23	5	0	− 1	0	1	43.23	45.13	− 1.89	85.63	86.04	− 0.41
22	6	0	1	0	− 1	8.77	9.83	− 1.06	70.58	70.06	0.52
18	7	1	0	− 1	0	13.10	13.95	− 0.84	61.31	61.92	− 0.62
17	8	− 1	0	− 1	0	33.01	35.08	− 2.07	52.50	52.69	− 0.19
13	9	0	− 1	− 1	0	29.26	28.28	0.99	58.57	58.81	− 0.24
11	10	− 1	0	0	1	19.13	18.95	0.18	75.80	75.40	0.40
15	11	0	− 1	1	0	47.39	47.60	− 0.21	90.64	89.97	0.68
2	12	1	− 1	0	0	50.27	49.02	1.25	92.76	92.07	0.69
27	13	0	0	0	0	26.35	27.87	− 1.52	65.87	68.35	− 2.48
6	14	0	0	1	− 1	17.16	16.49	0.67	85.12	85.84	− 0.72
25	15	0	0	0	0	29.79	27.87	1.92	67.52	68.35	− 0.83
29	16	0	0	0	0	30.18	27.87	2.31	69.11	68.35	0.76
16	17	0	1	1	0	3.18	3.42	− 0.23	70.64	70.16	0.47
26	18	0	0	0	0	25.49	27.87	− 2.38	68.75	68.35	0.40
20	19	1	0	1	0	32.39	33.28	− 0.89	82.87	82.57	0.30
8	20	0	0	1	1	35.96	33.18	2.77	76.22	77.68	− 1.46
21	21	0	− 1	0	− 1	29.17	30.57	− 1.40	68.54	69.31	− 0.77
4	22	1	1	0	0	0.31	− 0.63	0.94	55.20	55.61	− 0.41
7	23	0	0	− 1	1	25.05	23.52	1.53	70.20	69.82	0.37
3	24	− 1	1	0	0	26.92	25.96	0.96	72.51	73.55	− 1.03
19	25	− 1	0	1	0	17.03	19.14	− 2.11	76.13	75.40	0.73
28	26	0	0	0	0	27.54	27.87	− 0.33	70.51	68.35	2.16
9	27	− 1	0	0	− 1	36.87	35.09	1.78	59.30	59.27	0.03
24	28	0	1	0	1	11.18	12.74	− 1.56	65.54	64.65	0.89
5	29	0	0	− 1	− 1	22.18	22.75	− 0.57	51.46	50.34	1.11

Variable	Variable code	Coded and actual levels									
		− 1	0	1							
Methyl orange conc. (mg/L)	X_1_	20	40	60							
Algal biomass (g/L)	X_2_	3	5	7							
Initial pH level	X_3_	4	7	10							
Incubation time (min)	X_4_	30	60	90							

The maximum removal percentages of both MO and copper ions were obtained in the trial no. 12 with percent of 50.27% for MO and 92.76% for copper ions when methyl orange conc. was 60 mg/L, algal biomass as biosorbent was 3 g/L, pH 7 and incubation time was 60 min at room temperature. Whereas, the minimum MO removal percentage was obtained in trial no. 22 with percent of 0.31% when methyl orange conc. was 60 mg/L, algal biomass as biosorbent was 7 g/L, pH 7, and incubation time was 60 min. On the other hand, the minimum copper removal percentage was obtained in trial no. 29 with percent of 51.46% when methyl orange conc. was 40 mg/L, algal biomass was 5 g/L, pH 4, and incubation time was 30 min. The negative value for the predicted percentage of MO removal indicated in run 22 is the probability that the conditions used in this experimental run has a negative test result with a negative impact on the biosorption process.

### Multiple regression analysis and ANOVA for methyl orange removal

The results of Box–Behnken experimental design for methyl orange removal (%) were statistically analyzed and the results of ANOVA and multiple regression analysis were presented in Tables 2 and 3. The analysis includes the adjusted R^2^ value, the predicted R^2^ value, probability *P-*value, Fisher test (*F*-test), coefficient of determination (R^2^) and the coefficient values. Linear, quadratic and interactions effects of the selected 4 independent factors were also determined. The regression model includes four linear (X_1_, X_2_, X_3_, X_4_ ), four interactions (X_1_X_2_, X_1_X_3_, X_1_X_4_, X_2_X_3_, X_2_X_4_, X_3_X_4_), and four quadratics (X^2^_1_, X^2^_2_, X^2^_3_, X^2^_4_ ) (Table 2). If the regression model’s R^2^ value is closest to 1, this reflects the strength of the model and the best predictability of the response^28^. Table 2 shows that the value of R^2^ of the regression model is 0.9865; this means that 98.65% of the variability of MO removal can be explained by the model. On the other hand, the adjusted R^2^ value is 0.9730 which is very high verifying that the model is highly significant. The predicted-R^2^ value is 0.9388 which revealed a strong agreement with the adjusted R^2^ value of 0.9730. Hence, revealing a reasonable agreement between the predicted and experimental values. The predicted-R^2^ of the regression model reveals the effective predictable responses for the new experiments.

**Table 2:** Analysis of variance for biosorption of methyl orange dye by using *Fucus vesiculosus.*

Source of variance		Sum of squares	Degrees of freedom	Mean of square	*F*-value	*P*-value	Coefficient estimate
Model		4305.84	14	307.56	73.15	< 0.0001*	27.87
Linear effect	X_1_	36.71	1	36.71	8.73	0.0104*	− 1.75
	X_2_	2116.34	1	2116.34	503.32	< 0.0001*	− 13.28
	X_3_	8.65	1	8.65	2.06	0.1734	0.85
	X_4_	228.86	1	228.86	54.43	< 0.0001*	4.37
Interaction effect	X_1_X_2_	533.17	1	533.17	126.80	< 0.0001*	− 11.55
	X_1_X_3_	311.05	1	311.05	73.98	< 0.0001*	8.82
	X_1_X_4_	618.60	1	618.60	147.12	< 0.0001*	12.44
	X_2_X_3_	310.56	1	310.56	73.86	< 0.0001*	− 8.81
	X_2_X_4_	33.91	1	33.91	8.07	0.0131*	− 2.91
	X_3_X_4_	63.41	1	63.41	15.08	0.0017*	3.98
Quadratic effect	X_1_^2^	2.40	1	2.40	0.57	0.4622	− 0.61
	X_2_^2^	11.21	1	11.21	2.67	0.1249	− 1.31
	X_3_^2^	23.32	1	23.32	5.55	0.0336*	− 1.90
	X_4_^2^	25.60	1	25.60	6.09	0.0271*	− 1.99
Error effect	Lack of fit	41.72	10	4.17	0.97	0.5608	
	Pure error	17.15	4	4.29			
R^2^	0.9865	Std. dev	2.05				
Adj R^2^	0.9730	Mean	25.47				
Pred R^2^	0.9388	C.V. %	8.05				
Adeq precision	33.67	PRESS	267.09				

*F* Fishers's function, *P* level of significance, *C.V.* coefficient of variation.

* Significant values.

**Table 3:** Fit summary for Box–Behnken design results for biosorption of methyl orange dye by using *Fucus vesiculosus.*

Source	Sum of squares	*df*	Mean square	*F-*value	*P-*value *P*rob > *F*
**Lack of fit tests**					
Linear	1956.99	20	97.85	22.83	0.0039*
2FI	86.29	14	6.16	1.44	0.3925
Quadratic	41.72	10	4.17	0.97	0.5608
**Sequential model sum of squares**					
Linear vs mean	2390.56	4	597.64	7.27	0.0006*
2FI vs linear	1870.70	6	311.78	54.25	< 0.0001*
Quadratic vs 2FI	44.58	4	11.14	2.65	0.0776

Source	Standard deviation	R-squared	Adjusted R-squared	Predicted R-squared	PRESS
**Model summary statistics**					
Linear	9.07	0.5477	0.4723	0.2965	3070.74
2FI	2.40	0.9763	0.9631	0.9450	240.02
Quadratic	2.05	0.9865	0.9730	0.9388	267.09

* Significant values, *df* degree of freedom, *PRESS* sum of squares of prediction error, *2FI* two factors interaction.

Adequate precision level higher than 4 is preferable and implies the model reliability. The present model used for methyl orange removal had a reasonable precision value of 33.67 revealing the model reliability. The statistically analyzed results of methyl orange removal (%) shows that the coefficient of variation % (C.V. = 8.05%) has relatively low value, indicating that the performed experiments are highly precise^29^. The PRESS value is 267.09, the standard deviation value is 2.05 and the model's mean is 25.47 (Table 2). ANOVA of the quadratic regression model of methyl orange removal (%) indicates high significance of the quadratic model as confirmed by the very low value of probability (*P-*value˂ 0.0001) and the high *F*-value of 73.15. The non-significant lack of fit (*P*-value = 0.5608) indicates that the present results are consistent with the model. In this study, the variables displaying *P*-values below 0.05 were considered to have significant impacts^30^.

The interpretation of the correlation between the examined variables (Table 2) relied on the signals of variable coefficients and *P*-values. Basically, the correlation between the two factors could be negative or positive. Consequently, the positive coefficient sign reveals a synergistic influence, while the negative coefficient sign reveals an antagonistic impact. It's clear from the coefficients values (Table 2) that the initial pH level (X_3_) and incubation time (X_4_) had positive effects on methyl orange removal (%). Whereas the negative coefficients values of both methyl orange conc. (X_1_) and the algal biomass (X_2_) means that they exert a negative effects on methyl orange removal (%) from aqueous solutions by *Fucus vesiculosus* biomass in the tested range of the examined variables. It was obvious from the *P-*values that the linear coefficients of X_1_, X_2_ and X_4_, the interaction between X_1_X_2_, X_1_X_3_, X_1_X_4_, X_2_X_3_, X_2_X_4_, X_3_X_4_ and quadratic impacts of X_3_ and X_4_ had significant effects. Furthermore, linear coefficients of X_3_ the quadratic effects of X_1_, X_2_ (*P*-value equals 0.4622, 0.1249; respectively) had non-significant effects on the methyl orange dye removal by *F. vesiculosus*.

Table 3 display the fit summary for Box–Behnken design of methyl orange removal by *F. vesiculosus.* The fit summary applied to select the appropriate model for the experimental results (linear, 2FI or quadratic model). The appropriate model is chosen on the basis of significant model terms and non-significant lack of fit tests. The fit summary results demonstrated that appropriate model for methyl orange removal by *F. vesiculosus* biomass is the two factors interaction (2FI) model which is significant with a very small *P-*value < 0.0001. Lack of Fit Test for two factors interaction (2FI) (with *F*-value = 1.44 and *P-*value = 0.3925) and quadratic models (with *F*-value = 0.97 and *P-*value = 0.5608) of methyl orange removal percentages by *F. vesiculosus* biomass are non-significant (Table 3). Furthermore, the model summary statistics for methyl orange removal percentages by *F. vesiculosus* biomass quadratic model recorded the lower standard deviation of 2.05 and the highest R^2^ of 0.9865, adjusted R^2^ of 0.9730, but two factors interaction (2FI) model recorded the highest predicted R^2^ of 0.9450.

By using the coefficients (Table 2), the 2nd-order polynomial equation describing the correlation between methyl orange removal percentages by *F. vesiculosus* biomass (Y) regarding MO concentrations, algal biomass, and initial pH level and incubation time as the following:

1 $$\begin{aligned} {\text{Y }} &= \, + { 27}.{87} - {1}.{\text{75 X}}_{{1}} - {13}.{\text{28X}}_{{2}} + 0.{\text{85X}}_{{3}} + {4}.{\text{37 X}}_{{4}} - {11}.{\text{55 X}}_{{1}} {\text{X}}_{{2}} + {8}.{\text{82 X}}_{{1}} {\text{X}}_{{3}} + {12}.{\text{44 X}}_{{1}} {\text{X}}_{{4}} \\ &\quad- {8}.{\text{81 X}}_{{2}} {\text{X}}_{{3}} - {2}.{\text{91 X}}_{{2}} {\text{X}}_{{4}} + {3}.{\text{98 X}}_{{3}} {\text{X}}_{{4}} - 0.{\text{61 X}}_{{1}}^{{2}} - {1}.{\text{31 X}}_{{2}}^{{2}} - {1}.{9}0{\text{ X}}_{{3}}^{{2}} - {1}.{\text{99 X}}_{{4}}^{{2}} . \end{aligned}$$

where Y is the predicted value of methyl orange azo dye removal (%) and X_1_‒X_4_ are the coded values of methyl orange azo dye concentration, the algal biomass concentration, initial pH level, and incubation time; respectively.

### Multiple regression analysis and ANOVA for copper removal

The results of Box–Behnken experimental design for removal of copper were analyzed using multiple regression analysis and the results were presented in Tables 4, 5. Table 4 shows the regression model determination coefficient (R^2^) = 0.9934 which means that the variations in copper removal of 99.34% could be described by the model. In addition, the adjusted coefficient of determination (adj R^2^ value) of 0.9867 was relatively high and validated that the model was very significant. On the other hand, the predicted value of the determination coefficient (predicted R^2^ value) of 0.9772 is in an excellent agreement with the adjusted R^2^ values (0.9867), which revealed a well-fit between the predicted and observed values of copper removal percentages. The model used for this experiment is therefore ideal for predicting the removal percentage of copper at the tested levels of independent parameters. The adequate precision value for the current model is 46.27, revealing the model reliability. The mean value is 69.99, PRESS value is 75.40 and the standard deviation is 1.25 (Table 4). Meanwhile, the % of the coefficient of variation (C.V. = 1.79%) is low, indicating that the performed experiment have a high level of reliability and precision^29^.

**Table 4:** Analysis of variance for simultaneous biosorption of copper (II) ions by using *Fucus vesiculosus.*

Source of variance		Sum of squares	Degrees of freedom	Mean of square	*F*-value	*P*-value	Coefficient estimate
Model		3291.74	14	235.12	149.49	< 0.0001*	68.35
Linear effect	X_1_	201.77	1	201.77	128.28	< 0.0001*	4.10
	X_2_	319.78	1	319.78	203.31	< 0.0001*	− 5.16
	X_3_	1409.73	1	1409.73	896.28	< 0.0001*	10.84
	X_4_	96.15	1	96.15	61.13	< 0.0001*	2.83
Interaction effect	X_1_X_2_	683.43	1	683.43	434.51	< 0.0001*	− 13.07
	X_1_X_3_	1.06	1	1.06	0.68	0.4247	− 0.52
	X_1_X_4_	109.66	1	109.66	69.72	< 0.0001*	− 5.24
	X_2_X_3_	89.84	1	89.84	57.12	< 0.0001*	− 4.74
	X_2_X_4_	122.43	1	122.43	77.84	< 0.0001*	− 5.53
	X_3_X_4_	190.88	1	190.88	121.35	< 0.0001*	− 6.91
Quadratic effect	X_1_^2^	0.16	1	0.16	0.10	0.7566	0.16
	X_2_^2^	9.90	1	9.90	6.30	0.0250*	1.24
	X_3_^2^	0.83	1	0.83	0.53	0.4787	− 0.36
	X_4_^2^	55.73	1	55.73	35.43	< 0.0001*	2.93
Error effect	Lack of fit	9.77	10	0.98	0.32	0.9351	
	Pure error	12.25	4	3.06			
R^2^	0.9934	Std. dev	1.25				
Adj R^2^	0.9867	Mean	69.99				
Pred R^2^	0.9772	C.V. %	1.79				
Adeq Precision	46.27	PRESS	75.40				

*F* Fishers's function, *P* level of significance, *C.V.* coefficient of variation.

*Significant values.

**Table 5:** Fit summary for Box–Behnken design results of copper (II) ions removal by using *F. vesiculosus.*

Source	Sum of squares	*df*	Mean square	*F-*value	*P-*value *P*rob > *F*
**Lack of fit tests**					
Linear	1274.06	20	63.70	20.79	0.0047*
2FI	76.76	14	5.48	1.79	0.3037
Quadratic	9.77	10	0.98	0.32	0.9351
**Sequential model sum of squares**					
Linear vs mean	2027.44	4	506.86	9.46	< 0.0001*
2FI vs linear	1197.30	6	199.55	40.35	< 0.0001*
Quadratic vs 2FI	66.99	4	16.75	10.65	0.0004*

Source	Standard deviation	R-squared	Adjusted R-squared	Predicted R-squared	PRESS
Model summary statistics					
Linear	7.32	0.6118	0.5471	0.3956	2002.95
2FI	2.22	0.9731	0.9582	0.9283	237.73
Quadratic	1.25	0.9934	0.9867	0.9772	75.40

*df* degree of freedom, *PRESS* sum of squares of prediction error, *2FI* two factors interaction.

*Significant values.

ANOVA for the regression model of copper removal (%) indicates that the model is highly significant as is apparent from a very small probability value [*P-*value ˂ 0.0001] with the Fisher’s *F* test (*F-*value = 149.49) (Table 4). The *P*-values were used to assess the significance of each coefficient. The *P*-values showed that linear effects of X_1_, X_2_, X_3_, and X_4_ with probability values of < 0.0001 are significant. Also, the interactions effects between X_1_X_2_, X_1_X_4_, X_2_X_3_, X_2_X_4_ and X_3_X_4_ with probability values of < 0.0001 are also significant (Table 4). While, the interaction between X_1_X_3_ (methyl orange conc. and initial pH level) had a non-significant effect (*P-*value˃0.05). Additionally, the probability value implied that the quadratic impact of X_2_, X_4_ had a significant effects on the copper removal (*P*-value = 0.0250, < 0.0001 respectively), meanwhile X_1_, X_3_ had an insignificant effects on the copper removal using *F. vesiculosus.*

The negative coefficient value reflects an antagonistic relationship between the variables and the percent removal value, whereas positive coefficient value reflects a synergism between the variables and the percent removal value. Accordingly, the negative values of coefficients means that copper removal % by the biomass of *F. vesiculosus* is negatively affected by the effect of linear or mutual interactions between two parameters, as well as the quadratic effects. Whereas, the positive values of coefficients means that copper removal percentages by the biomass of *F. vesiculosus* are increased in the evaluated levels of the selected process parameters as affected by linear effects, mutual interactions effects or quadratic effects. It can be seen from the values of coefficients (Table 4) that X_1_, X_3_ and X_4_ had positive effects on copper removal % by the biomass of *F. vesiculosus*. However, X_2_ exerted negative effect on the copper removal % by the biomass of *F. vesiculosus*.

Table 5 indicated the results of the Fit summary of Box–Behnken experimental design of biosorption of copper by using brown alga *F. vesiculosus*. The fit summary results demonstrated that the quadratic model is the appropriate model for fitting copper removal by *F. vesiculosus* biomass with a very small *P-*value < 0.0004 and non-significant lack of fit (*P-*value = 0.9351 & *F*-value = 0.32). Furthermore, the model summary statistics of the quadratic model recorded the largest adjusted R^2^ (0.9867), predicted R^2^ (0.9772) and the lowest standard deviation (1.25).

By using the coefficients (Table 4), the 2nd-order polynomial equation describing the correlation between copper removal percentages by *F. vesiculosus* biomass (Y) regarding MO concentrations, algal biomass, and initial pH level and incubation time as the following:

2 $$\begin{aligned} {\text{Y }} &= \, + {68}.{35} + {4}.{1}0{\text{X}}_{{1}} - {5}.{\text{16X}}_{{2}} + {1}0.{\text{84X}}_{{3}} + {2}.{\text{83X}}_{{4}} - {13}.0{\text{7X}}_{{1}} {\text{X}}_{{2}} - 0.{\text{52X}}_{{1}} {\text{X}}_{{3}} - {5}.{\text{24X}}_{{1}} {\text{X}}_{{4}} \\ &\quad- {4}.{\text{74X}}_{{2}} {\text{X}}_{{3}} - {5}.{\text{53X}}_{{2}} {\text{X}}_{{4}} - { 6}.{\text{91X}}_{{3}} {\text{X}}_{{4}} + 0.{\text{16X}}_{{1}}^{{2}} + {1}.{\text{24X}}_{{2}}^{{2}} - 0.{\text{36X}}_{{3}}^{{2}} + {2}.{\text{93X}}_{{4}}^{{2}} . \end{aligned}$$

where Y is the predicted value of copper removal (%) and X_1_‒X_4_ are the coded values of methyl orange azo dye concentration, the algal biomass concentration, initial pH level, and incubation time; respectively.

### Three dimensional (3D) surface plots for MO dye and copper removal percentages

**Figure 1:**
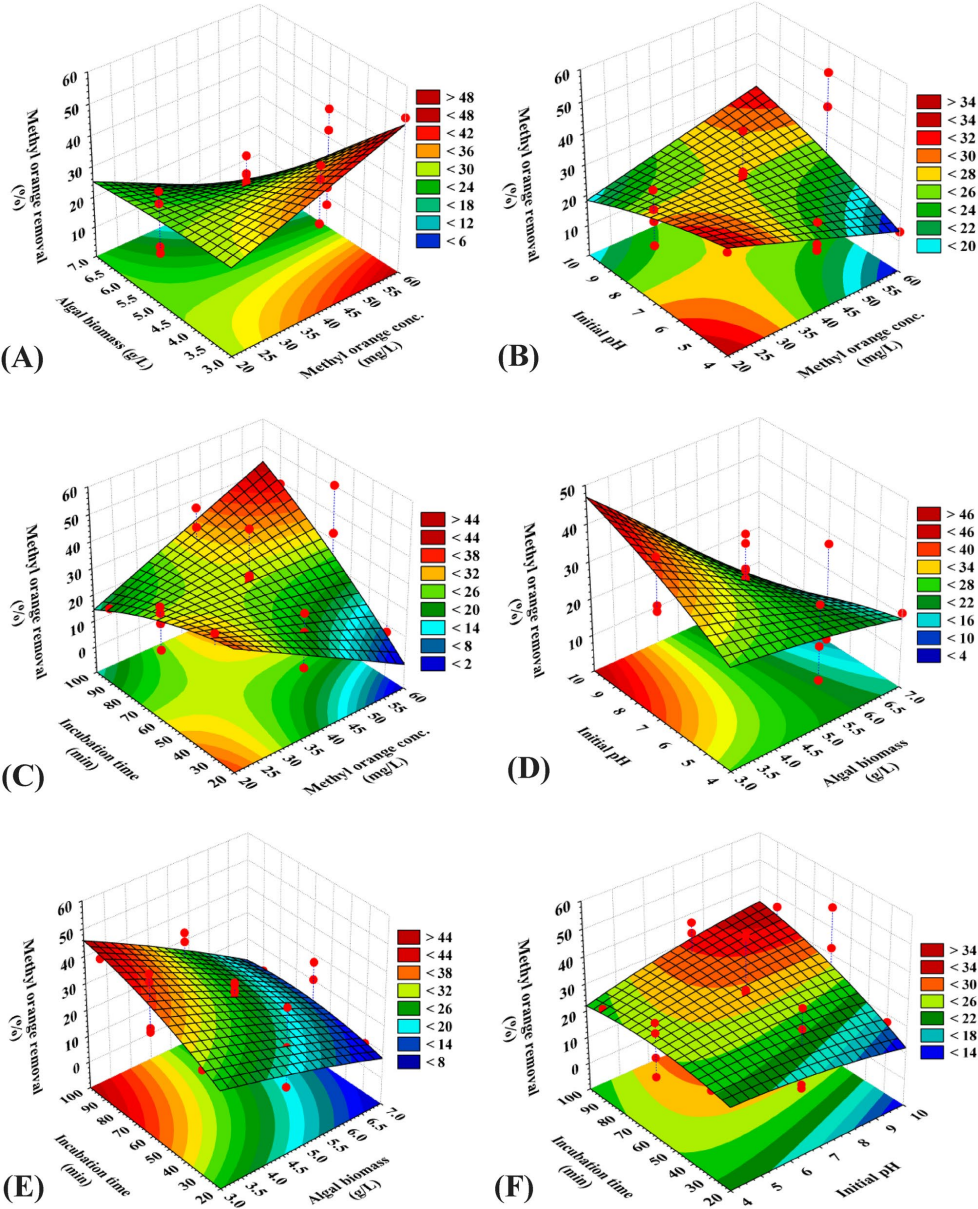
Three-dimensional surface plots of biosorption of methyl orange dye by *Fucus vesiculosus* biomass displaying the interactive effects of the four tested variables. This figure was created by using statistical software package, STATISTICA software (Version 8.0, StatSoft Inc., Tulsa, USA).

3D surface plots were created for determination of the optimum conditions of the bioprocess (removal of MO dye and copper ions from binary aqueous solution) and to describe the relationship between the methyl orange dye and copper removal percentages by *F. vesiculosus* biomass and the interactions between the chosen process variables. The 3D response surface plots were created for the pairwise of the four variables (X_1_X_2_, X_1_X_3_, X_1_X_4_, X_2_X_3_, X_2_X_4_ and X_3_X_4_). All experiments were conducted using a fixed concentration of copper ions of 200 mg/L. The removal percentages of MO dye were drawn on the Z-axis versus two variables, while the remaining variables maintained fixed at their zero levels (Fig. 1).

The 3D (Fig. 1A) shows the effect of MO concentrations (X_1_) and algal biomass (X_2_) on MO removal %, while the other two parameters (X_3_ and X_4_) are maintained fixed at their zero levels. The results have shown that the MO removal percentage was relatively high at higher MO concentrations and lower algae biomass. With the increase in the initial concentration of methyl orange dye from 20 to 57.62 mg/L, the removal percentage of methyl orange increases. The increasing percentage of removal could be mainly because of a larger surface area and the accessibility of unsaturated binding sites at the surface of *F. vesiculosus* biomass required to the biosorption process. Then, the removal percentage of methyl orange decreased with increasing concentration of methyl orange above 57.62 mg/L. This can be due to the saturation of binding sites on the surface of *F. vesiculosus* biomass. On the other hand, the removal percentage of methyl orange increases with the increase of initial concentration of *F. vesiculosus* biomass up to 3.2 g/L. The increased removal percentage of methyl orange could be mainly because of a larger surface area and the availability of unsaturated binding sites at the surface of *F. vesiculosus* biomass required to the biosorption process reaction by increased concentration of the algal biomass. The removal percentage of methyl orange then decreased with increasing the algal biomass concentration from 3.2 to 7 g/L. The agglomeration of the biomass can be a reason for the decrease in the removal efficacy.

Figure 1B show the effect of MO concentration (X_1_) and initial pH level (X_3_) on the MO removal percentage at center levels of alga biomass (X_2_) and incubation time (X_4_). Figure 1B indicates that the highest percentage of MO removal (61.97 mg/L) was at MO 57.7 mg/L and pH 9.5, when alga biomass was 3 g/L and incubation time was 60 min. The increase in the initial pH resulted in the highest percentage of orange methyl removal. This could be interpreted by the enhanced access of methyl orange to the active sites of the algal biomass at alkaline pH. Figure 1C show the effect of MO concentration (X_1_) and incubation time (X_4_) on the percentage of MO removal while alga biomass and pH were kept at their center points. The removal percentages of methyl orange increases with the increase of both initial concentration of methyl orange dye and incubation time. In addition, the effects of brown alga *F. vesiculosus* biomass (X_2_), initial pH level (X_3_) and incubation time (X_4_) on the MO removal percentage are also presented in Fig. 1D–F.

**Figure 2:**
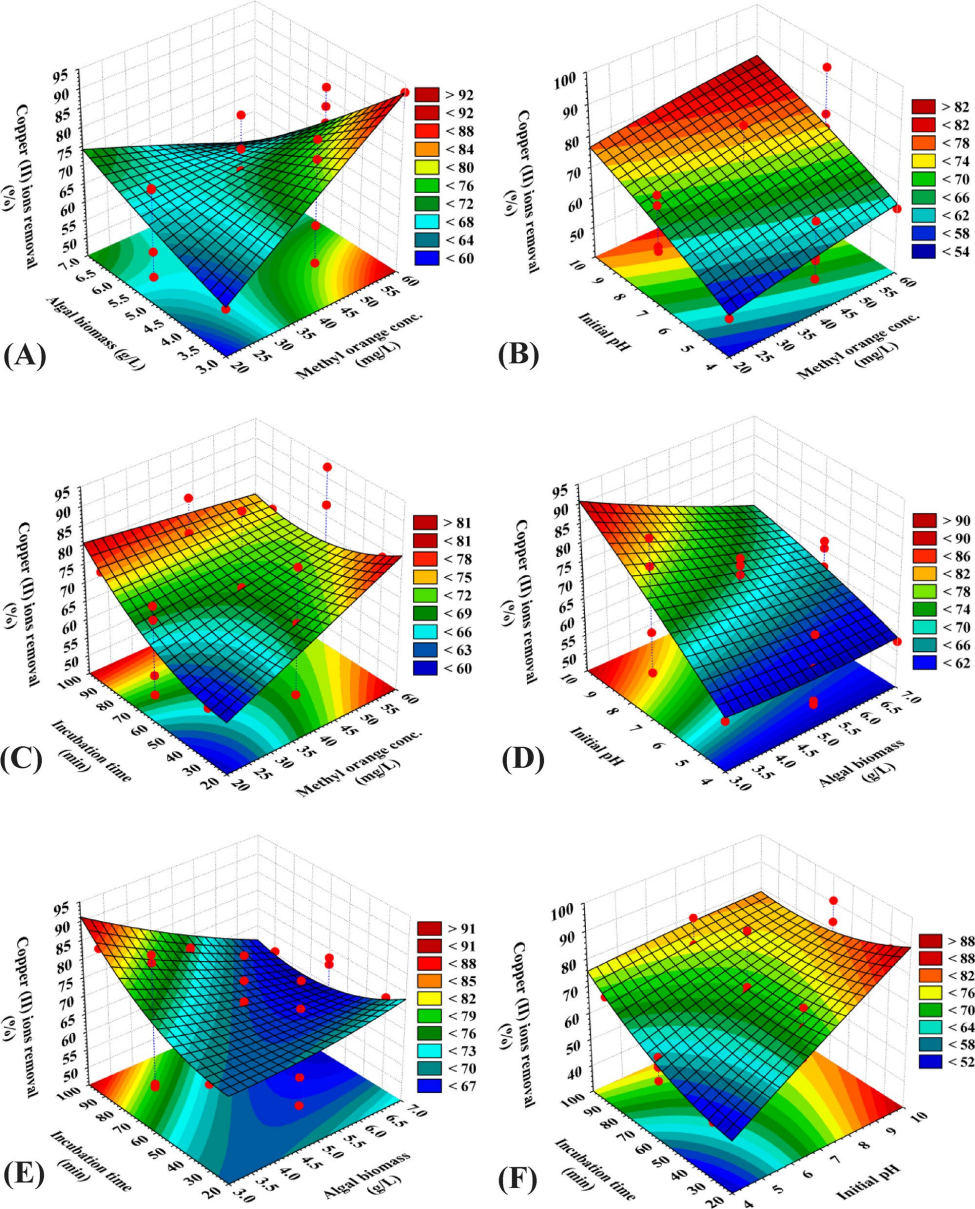
Three-dimensional surface plots of biosorption of copper (II) ions by *Fucus vesiculosus* biomass displaying the interactive effects of the four tested variables. This figure was created by using statistical software package, STATISTICA software (Version 8.0, StatSoft Inc., Tulsa, USA).

Similarly, the three-dimensional plots (Fig. 2) represent the effects of MO concentration, brown alga *F. vesiculosus* biomass, initial pH level and incubation time on copper removal percentage. Figure 2A–C indicated that the higher level of MO concentration increases the percentage of copper removal by brown alga *F. vesiculosus* biomass in aqueous solution. The 3D plots of Fig. 2D,E indicated that lower levels of brown alga *F. vesiculosus* biomass causes higher removal of copper. In addition, the effects of initial pH level (X_3_) and incubation time (X_4_) on copper removal percentage are also presented in Fig. 2F.

### Effect of initial pH value on the biosorption process

pH is a significant factor that influences the degree of ionization and the characterizations of biosorbent surface^31^. As the pH increases, the pH value leads to an increase in biosorption capacity of heavy metals ions; this may be caused by a decline of the competitiveness between positively charged metal ions and H^+^^32^. As the pH increases, OH^−^ and anionic dyes competing with each other and dyes uptaking were lower, the optimal pH was 6 for removal of Ni^2+^ and Zn^2+^ and pH was 4 for removal methyl orange^33^. In acidic conditions the electrostatic attractions take place between anionic dye (negatively charged) and biosorption sites of the adsorbent (with a positive charge), causing an increase in the biosorption capacity^34^. The current study proved that the optimum pH of Cu^2+^ and methyl orange simultaneous removal was 7.

The effect of pH on MO and Cu removal can be described as an electrostatic interaction mechanism between adsorbents and alga surface; at lower pH, a competition between anionic dyes and protons found in the active sites of the biosorbent, thus the biosorption of the anion dyes is not optimal^35^. Increasing the solution pH reveals increasing the biosorption of anionic ions because the elevated of electrostatic interactions between anionic dyes and adsorbent ^36^. The enhanced biosorption at higher pH values could be due to the negatively charged OH^‒^ functional groups that are present on the surface of the biosorbent. This results in electrostatic attraction between the cationic dyes and negatively charged biosorbent surface^37^.

### Effect of biomass concentration on the biosorption process

Algae biomasses are considered to be effective sustainable biosorbents for large-scale uses for metals and hazardous dyes biosorption due to their cell wall constitutes high metal removal efficiency, renewable and cost-effective^38^. The metal-binding ability of each alga was different. This can be clarified by the variation in polysaccharides and proteins' cell wall composition that provides cell surface binding sites. Schiewer and Volesky^39^ claimed that the high biosorption capacity of algal surface could be attributed to the existence of polysaccharides, lipid or proteins molecules in their cell walls that containing functional groups which can act as binding sites for metals. The biosorption capacity of the algal surface is attributed to the availability of the functional groups, for example carboxylic, hydroxyl, phosphate, imidazole, sulfate, sulphuryl, phosphoryl, amino, etc^40^.

In the present study, increase in the removal percentages of Cu^2+^ and methyl orange with increasing the biomass concentration could be attributed to the increase in the surface area of brown marine alga *Fucus vesiculosus* biomass and the availability of more active sites. Phugare et al.^41^ stated that an improvement in the biosorption percentage with increasing biomass concentration is anticipated due to the increased biosorbent surface area which in turn increases the number of active sites leading to efficient biosorption. On the other hand, a reduction in the removal percentages at higher biomass concentrations of brown marine alga, *Fucus vesiculosus*, could be attributed to the agglomeration of the biomass. Karthikeyan et al.^42^ stated that a reduction in the efficiency of the removal at higher concentrations of the algal biomass could be caused by a reduction in the efficiency of the biomass surface area as a result of agglomeration. Furthermore, Lata et al.^43^ documented a reduction in the biosorption potential at greater algal concentrations was due to agglomerations of the biomass, which could in turn reduce the intercellular spacing which reduced the overall effective biosorption surface area, thus reducing the number of active binding sites available on the algal biomass surface. However, EL Hassouni et al.^44^ concluded that the decrease in the efficiency of the biosorption process after achieving the optimal dose could be attributed to an increase in the number of unsaturated active sites on the biosorbent surface with an increase in the concentration of biomass because of the ineffective utilization of the active sites where metal ions or adsorbate particles are insufficient to bind to all available active sites.

Kumar et al.^45^ reported that 0.1 g of *Ulva fasciata* is adequate to eliminate 95% of copper from aqueous solution. The biosorption of copper was maximum by using 2 g of *Callithamnion corymbosum* sp.^46^. Abdulkareem & Alwared^47^ reported that the increase of alginate beads derived from marine algae above 10 g/L resulted in a decrease of the biosorption processes which could be attributed to an increase in the number of unsaturated active sites on the biosorbent surface. The effective concentration of brown macro-marine algae *Gelidiella acerosa* as biosorbent was 0.41 g/L to remove 96.36% of copper from aqueous solution^48^. The highest removal value of copper ions (88.45%) was obtained when 4 g/L of marine brown alga *Sargassum bevanom* was applied as biosorbent when pH was adjusted to 6, and incubation time of 100 min^49^. The removal of copper is performed effectively by some marine algae such as *Fucus vesiculosus* by using 1.85 mmoL/g^21^.

The greatest removal percentage of methyl orange was 97% when using 0.4 g/L *Oedogonium subplagiostomum* AP1 biomass as biosorbent^50^. Five mg of the activated carbon of marine alga *Gracilaria corticata* has the ability for decolourization of textile dye^51^. The maximum dye decolorisation percentage (86.1%) by calcium alginate extracted from *Sargassum* sp. was obtained by using 40 mg/L alginate^52^.

### Effect of contact time on the biosorption process

In the present study, the simultaneous removal of cationic copper ions and anionic methyl orange azo dye by brown marine alga *Fucus vesiculosus* biomass from binary solution depends on the contact time. Experimental results have shown obviously that the removal percentage of cationic copper ions and anionic methyl orange azo dye increases as the contact time increase up to the optimum, which probably due to the availability of a large number of surface vacant active sites on the brown marine alga *Fucus vesiculosus* biomass surface and also cationic copper ions and anionic methyl orange azo dye concentrations were high. However, at higher contact time, the active sites were saturated causing no further adsorption occurs. Saturation of all active sites on the biomass surface results in a state of equilibrium^53^.

Biosorption capacity of heavy metals by brown algae increased with increasing contact times, within 60 min, the absorption of nickel and cadmium reached 95%^54^. Biosorption of Fe^3+^ by *Sargassum vulgare* (brown alga) was elevated with increasing the time up to 50 min^55^. Uptake of methyl blue by *Sargassum muticum* was fast in the first 5 min and reach equilibrium within 60–90 min^56^. The elimination of dye reached 93% at 45 min. by *Sargassum crassifolium*^57^.

### Desirability function (DF)

The key objective of the experimental design and the desirability function (DF) were used to identify the optimum predicted conditions to maximize the responses^58^. The DF values ranged between zero (undesirable) to one (desirable). The numerical optimization defines the points minimizing the desirability function. For the optimization process, the DF option in the software design expert (Version 7.0.0) was used.

**Figure 3:**
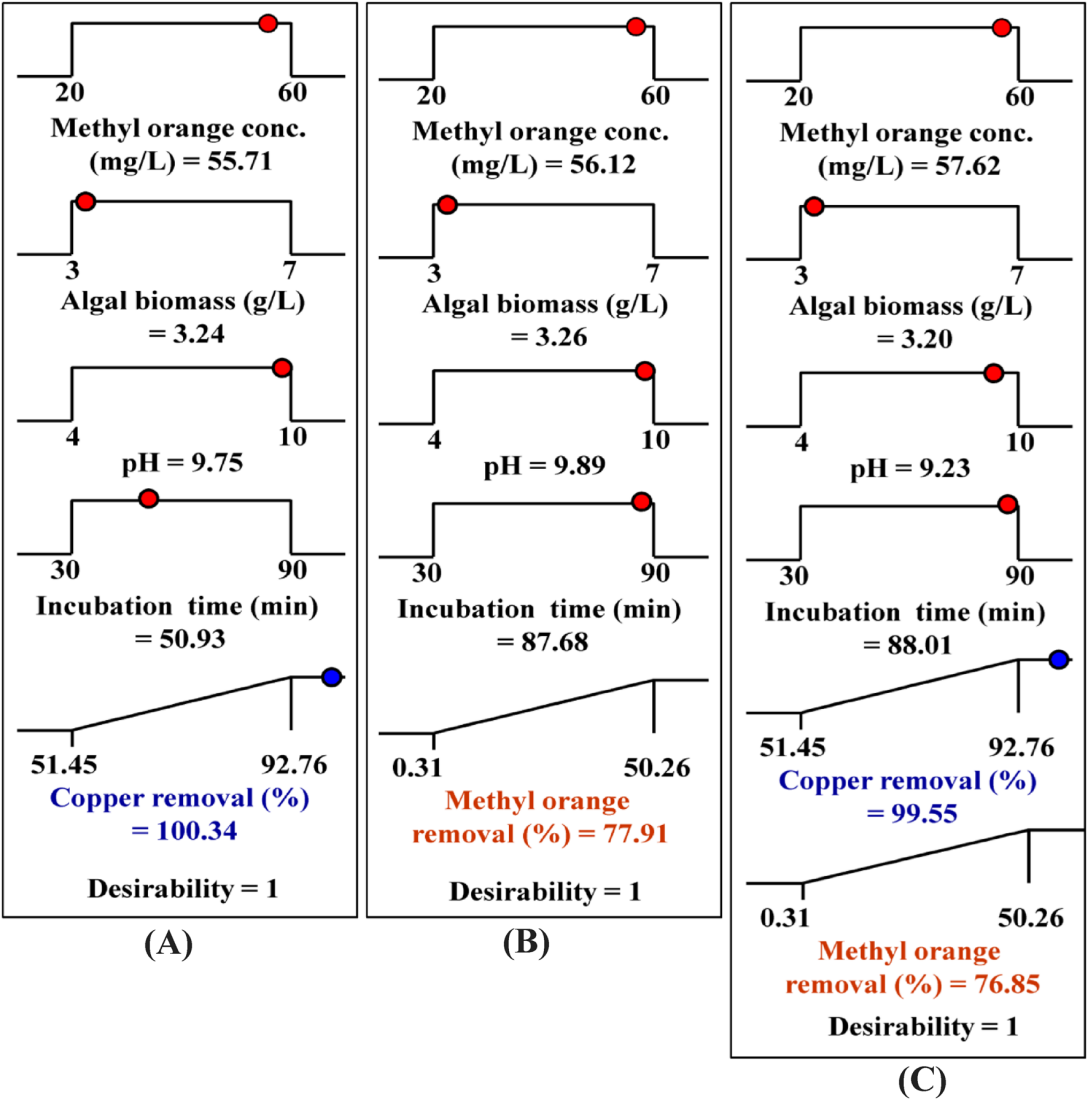
The optimization plot displays the optimum predicted values and the desirability function for maximum simultaneous removal percentages of anionic methyl orange azo dye as mono-component **(A)**; cationic copper ions as mono-component **(B)**; both copper ions and methyl orange in binary multi-component system **(C)**. This figure was created by using Design Expert version 7 for Windows software.

Figure 3 shows the optimization plot displays the optimum predicted values and the desirability function for maximum removal percentage of cationic copper ions and anionic methyl orange azo dye by brown marine alga *Fucus vesiculosus*.

Maximum removal percentage of cationic copper ions as mono-component by brown marine alga *Fucus vesiculosus* (100.34%) was obtained by using methyl orange azo dye concentration of 55.71 mg/L, the algal biomass concentration of 3.24 g/L, initial pH level of 9.75, and incubation time of 50.93 min. Whereas, the optimal predicted conditions attained for the maximum removal percentage of anionic methyl orange azo dye as mono-component (77.91%) were methyl orange azo dye concentration of 56.12 mg/L, the algal biomass concentration of 3.26 g/L, initial pH level of 9.89, and incubation time of 87.68 min. On the other hand, the simultaneous removal percentages of cationic copper ions and anionic methyl orange azo dye in multi-component system by brown marine alga *Fucus vesiculosus* were obtained using methyl orange azo dye concentration of 57.62 mg/L, the algal biomass concentration of 3.20 g/L, initial pH level of 9.23, and incubation time of 88.01 min. The previous predicted conditions for simultaneous removal of copper ions and methyl orange azo dye by brown marine alga *Fucus vesiculosus* could be resulted in maximum removal percentages of 99.55% for copper and 76.85% for methyl orange azo dye with DF of 1.

In order to verify the removal percentages of cationic copper ions and anionic methyl orange azo dye by brown marine alga *Fucus vesiculosus* under the optimal predicted conditions, the experiments have been done in triplicate and compared with the predicted values. The experimental results for copper ions and methyl orange azo dye removal percentages were 97.7% and 75.49; respectively. The verification showed that the experimental results and their predicted values of a strong agreement imply that the DF effectively determines the optimal predicted conditions for the simultaneous removal of cationic copper ions and anionic methyl orange azo dye by brown marine alga *Fucus vesiculosus*.

### FTIR analysis

**Figure 4:**
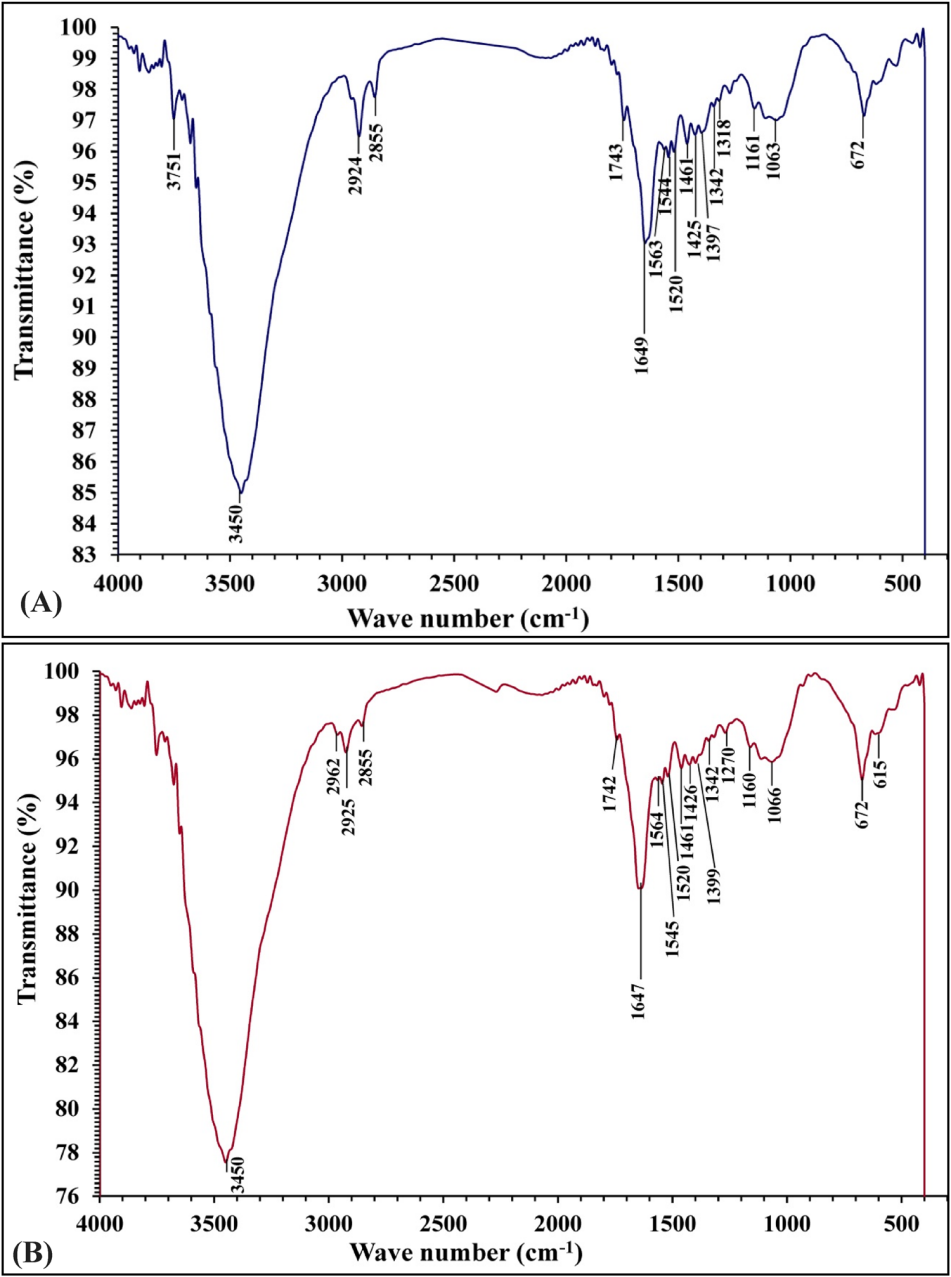
FTIR of *F. vesiculosus* before **(A)** and after **(B)** MO and copper ions biosorption from aqueous solution.

The FTIR spectrums of brown alga *F. vesiculosus* biomass were analyzed before and after bio-adsorption of methyl orange and copper (Fig. 4) showing differences due to the interaction of methyl orange and copper ions with active sites (functional groups) that found in cell surface in biomass. The brown algae cell walls consist of cellulose, hemicelluloses, sulphated furans and also some unique polysaccharides (alginates) that have many active groups (hydroxyl, carboxylate, amino and phosphate groups) with negative charges that can interact with cationic dye and connect the ions of heavy metal^59^. The spectrum of FTIR analysis of brown alga *F. vesiculosus* before and after bio-sorption of MO and copper ions showed different absorption peaks at 3751, 3450, 2924, 2855, 1743, 1649, 1563, 1544, 1520, 1461, 1425,1397, 1342,1318, 1161,1063 and 672 cm^−1^ which has been shifted to 3450, 2962, 2925, 2855, 1742, 1647, 1564, 1545, 1520, 1461, 1426, 1399, 1342, 1270, 1160, 1066, 672 and 615 cm^−1^. Figure 4 represent that very narrow peak at 3751 are present in the brown alga *F. vesiculosus* and no present after absorption MO and copper ions. Some other peaks are shifted in range 3 to –22 cm^−1^ due to the brown alga adsorbed copper ions and methyl orange. Also, the new peaks were observed at 1270 and 615 cm^−1^ this change may be due to bind of MO and copper on algal biomass. The narrow peak at 3751 cm^−1^ and broad peak at 3450 cm^−1^ are specified to the elongation of O–H group such as phenols, carboxylic acids, and alcohols^60^. Theivandran et al.^61^ stated that peeks at 2924 cm^−1^, 2962 cm^−1^ and 2855 cm^−1^ are related to C–H protraction vibration presence of alkenes. Peaks at 1743 and 1742 cm^−1^ are related to C=O stretching mode of lipids^62^. Peaks at 1649 cm^−1^ and 1647 cm^−1^ are correlated to N–H of adenine, thymine, guanine, cytosine^63^, peaks at 1544 cm^−1^, 1545 at cm^−1^ and 1520 at cm^−1^ are correlated to amide II bands arises from C–N stretching^64–66^. Peak at 1461 cm^−1^ is correlated to C–H bend elongation vibration presence of alkenes^67^, peaks at 1425 cm^−1^ and 1426 cm^−1^ are associated to C–C elongation (in–ring) aromatics^68^, peaks at 1397, 1399 are associated to CH_3_^69^, peaks 1342 and 1318 cm^−1^ are represent C–H bending^70,71^, while peaks at 1270 C–C–O stretching^72^, peaks at 1161 and 1160 cm^−1^ represent stretching vibrations of hydrogen–bonding C–OH groups^73^. Peaks at 1063 and 1066 interrelated to PO_2_ symmetric stretching of nucleic acids^74^.

### Scanning electron microscopy

**Figure 5:**
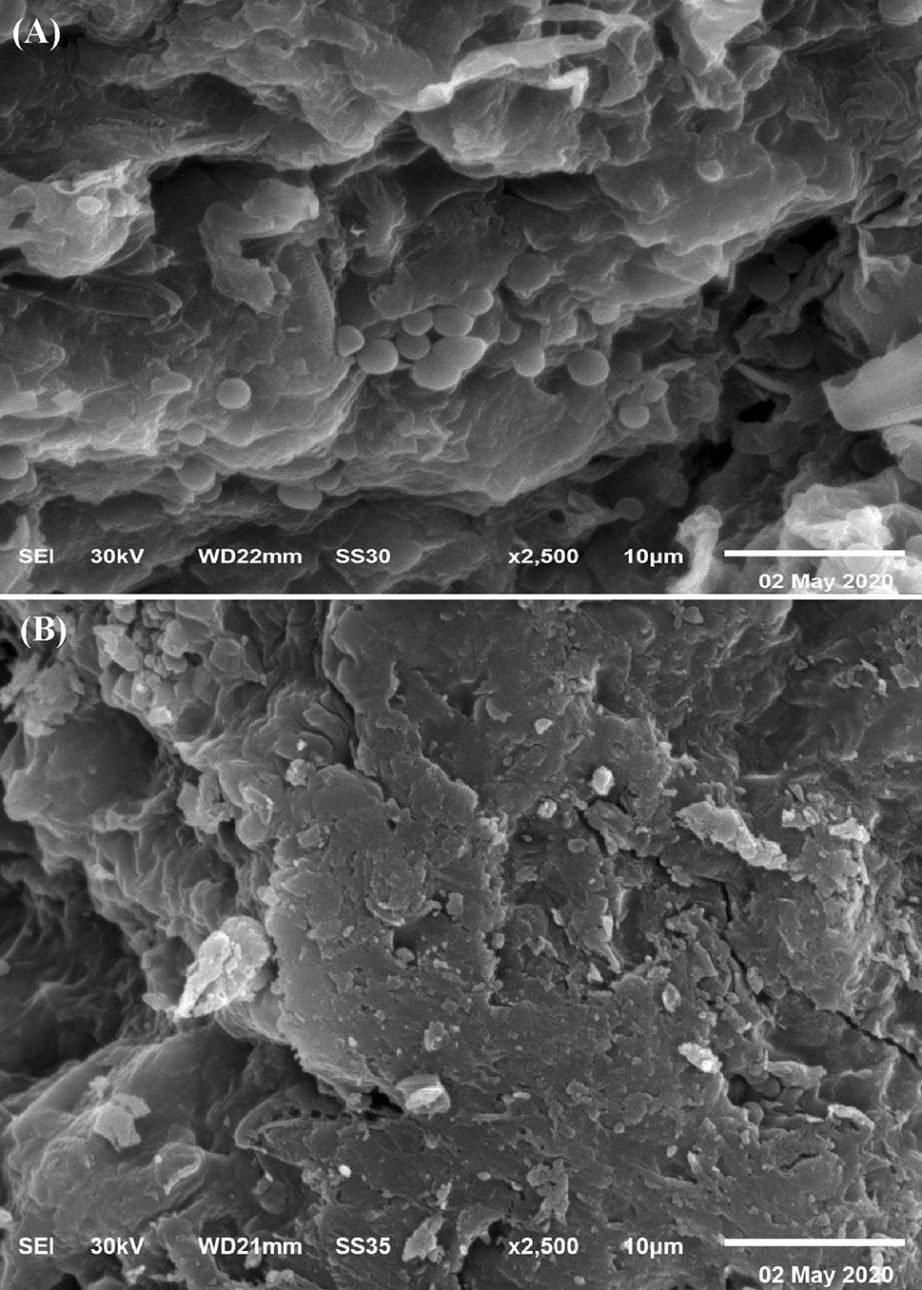
SEM micrograph of *F. vesiculosus* **(A)** before and **(B)** after biosorption of MO and copper ions from aqueous solution.

The SEM images demonstrated that the dry brown alga *F. vesiculosus* biomasses after and before the biosorption of MO and copper ions as shown in Fig. 5A,B. Figure 5B indicated the ability of *F. vesiculosus* to biosorb MO and copper ions. After biosorption of MO and copper ions, the surface of *F. vesiculosus* biomass has been more shrinking, irregular, and there are also more glossy spots as a result of accumulation of copper ions on the cell surface^75^. The morphological structure of the algae changed after biosorption of methyl orange by *Oedogonium subplagiostomum* ^50^.

### Electron dispersive spectroscopy (EDS)

**Figure 6:**
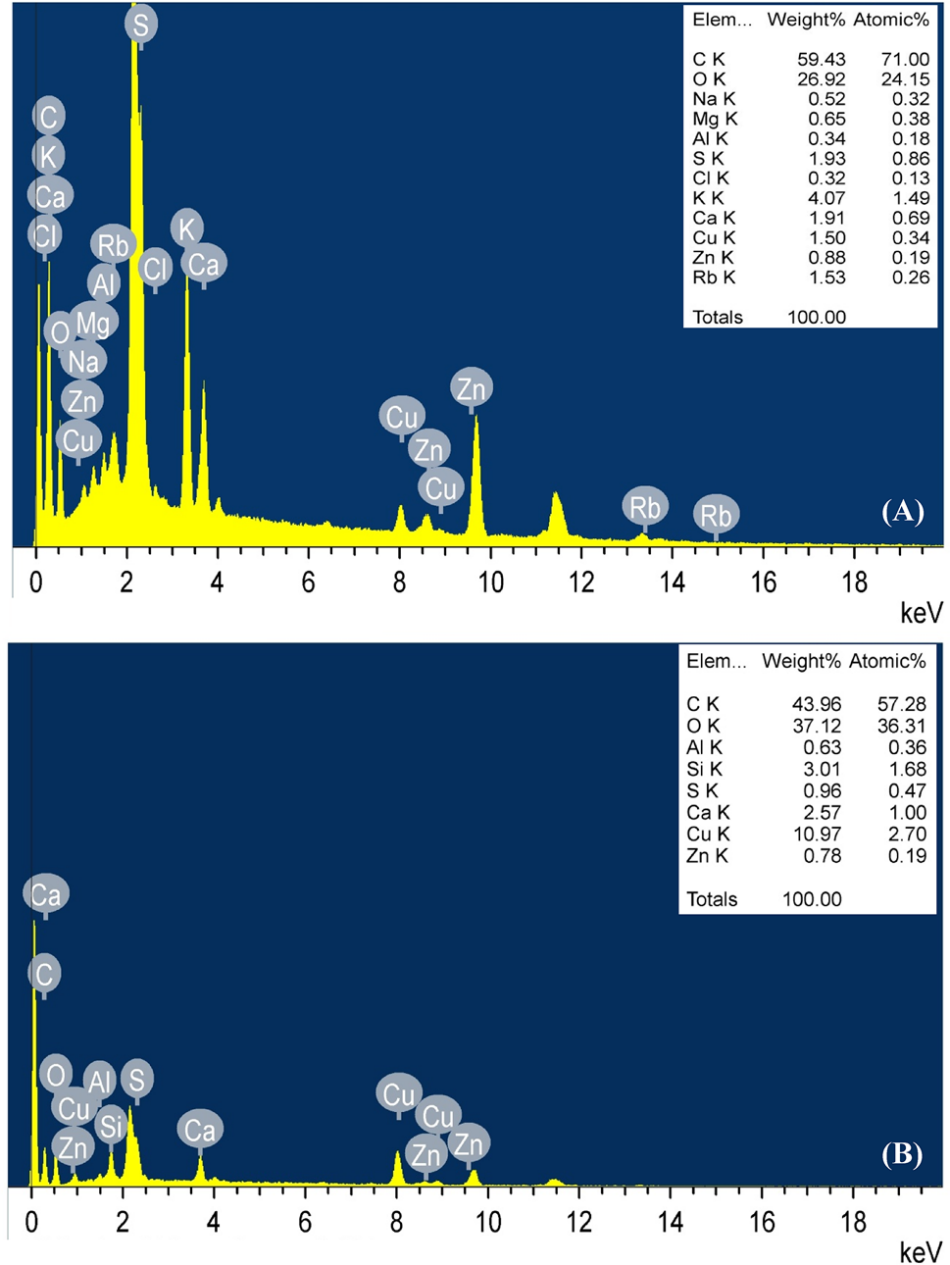
EDS micrograph of *F. vesiculosus* **(A)** before and **(B)** after biosorption of MO and copper ions from aqueous solution.

The EDS analysis is analytical technique used for the chemical characterization or both qualitative and quantitative elemental analysis of a sample surfaces^76^. In this experiment, EDS analysis was carried out to find the elements present on the surface of *F. vesiculosus* biomass and verified the attachment of Cu^2+^ to the surface of *F. vesiculosus* biomass after biosorption process. The EDS-TEM analysis (Fig. 6A) indicated the existence of optical absorption peak corresponding to Cu^2+^ before the biosorption process which could be due to the TEM copper grids coated with a carbon foil have been used during the analysis. The EDS spectra (Fig. 6B) reveal the presence of an optical absorption peak corresponding to Cu^2+^ after biosorption process attached to *F. vesiculosus* biomass cell surface. The Cu^2+^ weights` were 1.5 and 10.97% before and after the biosorption process; respectively that proves the role and capacity of *F. vesiculosus* biomass in the biosorption process of Cu^2+^ from aqueous solutions.

### Mechanisms of biosorption

Raize et al.^77^ observed that biosorption of the metallic cations to the algal cell wall components was a surface process. Biosorption by algal biomass occurs mainly through cell wall interactions^78^. Brown algae biomass cell walls contain many polymers including high concentrations of polysaccharides (sulfated polysaccharides, alginate) and proteins which involve several functional groups^77^. There are many functional groups on the algal cell wall such as carboxyl, sulphate, hydroxyl, carboxyle and amino groups, which can serve as cell surface binds for contaminants.

**Figure 7:**
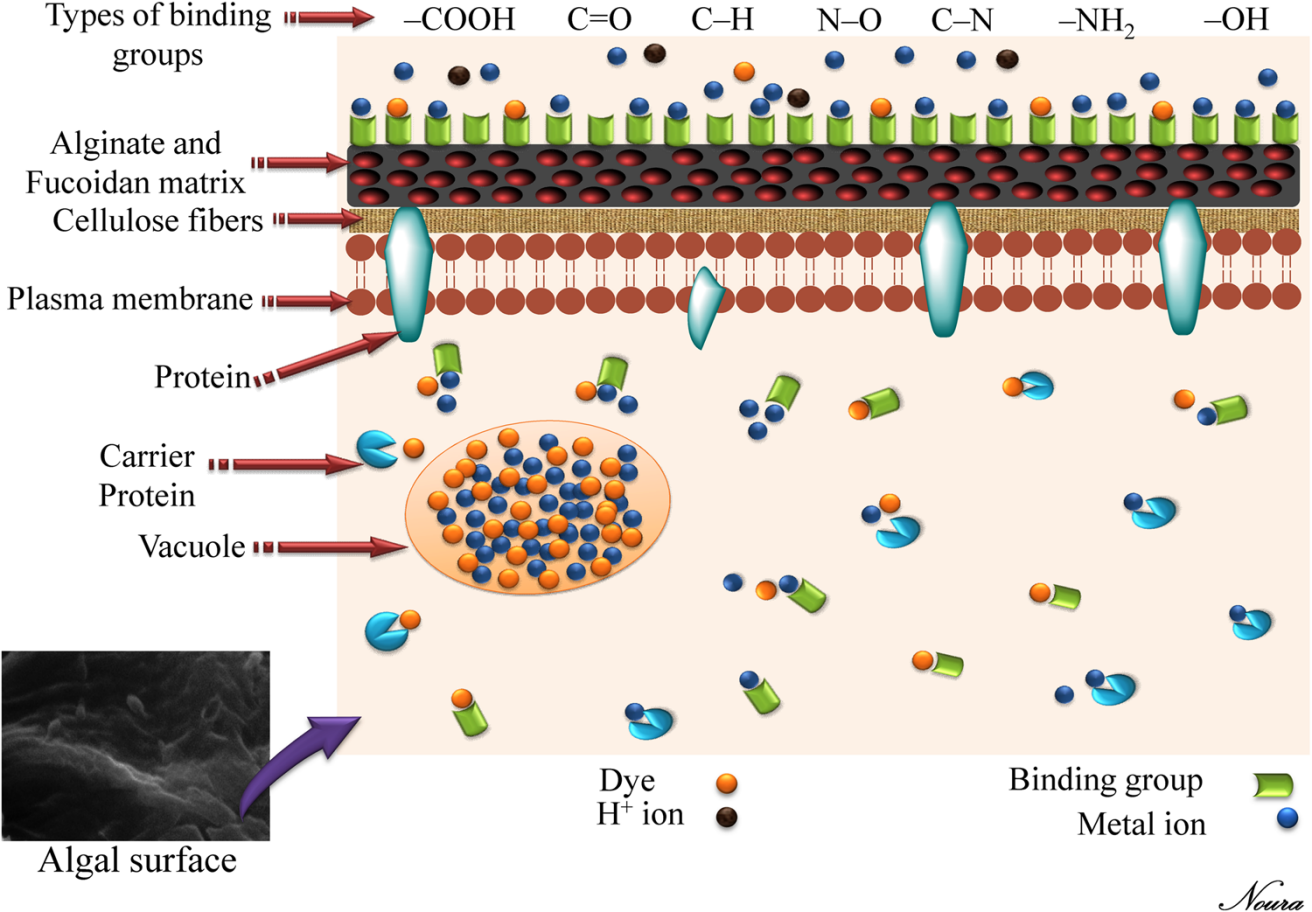
Biosorption mechanism of metal and dye by algal biomass.

Biosorption of metals involves several mechanisms that differ qualitatively and quantitatively according to the species used, the origin of the biomass, and its processing procedure. The principal binding mechanisms of the biosorption process by the algal biomass include ion exchange, formation of complex between contaminants cations and the ligands on the algal surface, diffusion interior the cells or surface precipitation, chelation, bioaccumulation within the cells, binding to intracellular components and proteins^19,79^ (Fig. 7) and reduction reactions, accompanied by metallic precipitation on the cell wall matrix^77^. Ion-exchange is a vital concept in the biosorption process, since it reflects the fact that most brown algal biomass is either protonated (ion-exchange takes place between various ions and protons at the biomass binding sites) or contains light metal ions such as K^+^, Na^+^ and Mg^2+^, which are released upon binding of a heavy metal cation with alginate^80^. Saturation of all active adsorption sites on the biomass surface results in a state of equilibrium^53^. The ion exchange capacity of the brown algae is directly related to the unique macromolecular structure of alginate that contains carboxylic groups which is the most abundant acidic functional group present in the alginate polymer polysaccharides^80^. The sulfonic acid of fucoidan is the second most common functional acidic group in brown algae. Sulfonic acid groups usually play a secondary function, except when metal binding occurs at a low pH level. Hydroxyl groups are also found in all polysaccharides but they are less concentrated and charged negatively only at pH > 10. This means they play a secondary role in metal binding at low pH^80^. Jang et al*.*^81^ acknowledged that guluronic acid-rich alginates (Na-alginate gel) display a high metal selectivity for Cu^2+^ due to its higher contents of guluronic acid residues.

## Materials and methods

### Preparation of the biosorbent

*Fucus vesiculosus* was collected from Jeddah beach, Saudi Arabia in April 2019. The *F. vesiculosus* biomass was washed carefully by using tap water, then by distilled water to get rid of salts and sand. After cleaning, the alga biomass was subjected to dryness at 50 °C, (up to steady weight). The dried alga was crushed and then sieved using a suitable laboratory sieve with a particle size range of 1–1.2 mm. The alga biomass was then used as biosorbent for simultaneous methyl orange and copper ions removal.

### Preparation of methyl orange and copper solutions

**Figure 8:**
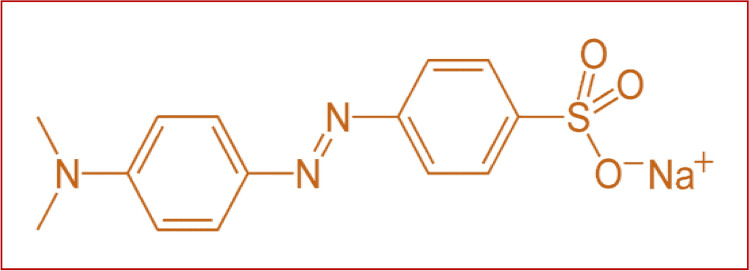
Methyl orange chemical formula.

The solutions required for the biosorption experiments were prepared by dissolving of weighed copper sulphate (Copper(II) sulfate anhydrous, powder, ReagentPlus, ≥ 99.0%) at a concentration of Cu^2+^, 200 mg/L and methyl orange at a work concentrations of 20, 40, 60 mg/L in distilled water. The desired initial pH level of each solution has been achieved by adding NaOH (0.1 N) or HCl (0.1 N). Methyl orange (MO) azo dye used in this study was obtained from Sigma-Aldrich Chemical Company, Inc., USA without any further purification (soluble in distilled water). The chemical formula of MO is C_14_H_14_N_3_O_6_S^−^ and the chemical formula is shown in Fig. 8. MO is an anionic azo organic compound that turns red in acidic medium and orange in basic medium.

### Optimization of the copper ions and methyl orange removal by batch biosorption using Box–Behnken design

Four factors were chosen to determine the optimum conditions for maximum simultaneous removal of copper ions and MO using the Box–Behnken design^29^. Design-Expert software (version 7) for Windows was used for generating the Box–Behnken design with 3 center points and 29 different experiments for predicting the optimum levels for the significant factors and to achieve the maximum simultaneous removal of copper ions and methyl orange by *Fucus vesiculosus* biomass. These factors were methyl orange concentrations (X_1_; 20, 40, 60 mg/L), algal biomass (X_2_; 3, 5, 7 g/L), initial pH (X_1_; 4, 7, 10) and incubation time (X_4_; 30, 60, 90 min). The tested variables were evaluated at 3 coded levels (+ 1 for high level, 0 for middle, and − 1 for low level). The biosorption experiments were carried out by applying batch biosorption experiments in 250 mL Erlenmeyer flasks and the working solution was 100 mL. The experimental studies were made in multi component system (binary solution). All experiments were carried out using a fixed concentration of copper ions of 200 mg/L and stirring at 200 rpm at ambient temperature.

The correlations between the selected variables of the biosorption process and the responses (copper ions and methyl orange removal percentages) were determined using the equation of second-degree polynomial as follows:

3 $$Y = \beta_{0} + \sum\limits_{i} {\beta_{i} } X_{i} + \sum\limits_{ii} {\beta_{ii} } X_{i}^{2} + \sum\limits_{ij} {\beta_{ij} } X_{i} X_{j}$$

In which Y is the predicted copper ions or methyl orange removal percentages, X_i_ is the coded levels of the selected variables, β_i_ (linear coefficient), β_ij_ (interaction coefficients) , β_0_ (regression coefficients) and β_ii_ (quadratic coefficients).

### Analytical methods

After the defined time, ten mL of the binary solution for each trial was centrifuged at 6000×*g* and the supernatants were analyzed by measuring the absorbance changes on a UV/Vis spectrophotometer at a wavelength of λ_max_ that was 467 nm, to determine the final (residual) concentrations (C_f_) of methyl orange dye. The efficiency of *Fucus vesiculosus* biomass for removal of methyl orange from aqueous solutions was calculated quantitatively in percentage by using the following equation:

4 $${\text{Methy}}\quad {\text{orange}}\quad {\text{elimination}} (\%) = \frac{{{\text{C}}_{{\text{i}}} - {\text{C}}_{{\text{f}}} }}{{{\text{C}}_{{\text{i}}} }} \times 100$$

where: C_f_, C_i_ are the final and initial concentrations of MO (mg/L); respectively.

Another 10 mL of the binary solution for each trial was centrifuged at 6000×*g* and the supernatants were analyzed for determination of the residual concentration of Cu^2+^ using Atomic absorptions (Buck scientific 2 hydrous system series Atomic Absorption (USA) by air acetylene system) at the Biotechnology Unit, Mansoura University Egypt according to “standard methods for the examination of water and wastewater 23rd edition 2017” ^82^. The efficiency of *F. vesiculosus* biomass to get rid of Cu^2+^ from hydrous solutions was detected in percentage utilizing the following equation.

5 $${\text{Copper}}\quad {\text{ions}}\quad {\text{removal}} (\%) = \frac{{{\text{C}}_{{\text{i}}} - {\text{C}}_{{\text{f}}} }}{{{\text{C}}_{{\text{i}}} }} \times 100$$

where: C_f_, C_i_ are the final and initial copper ions concentrations (mg/L); respectively.

All evaluations of both Cu^2+^ and MO in the binary solutions were estimated in triplicate.

### Statistical analysis

Design Expert version 7 (https://www.statease.com/software/design-expert/) and STATISTICA version 8 (https://www.statsoft.de/de/software/statistica) softwares have been used for the generation of the experimental design, statistical analysis and to draw the three-dimensional surface plots.

### Fourier transforms infrared (FTIR) spectroscopy

The dry biomass of *F. vesiculosus* samples was analyzed using FTIR spectroscopy before and after methyl orange and copper ions removal. The samples of dry biomass were mixed with pellets of potassium bromide and the FTIR spectra were then detected between 400–4000 cm^−1^ using "Thermo Fisher Nicolete IS10, USA spectrophotometer".

### Scanning electron microscopy (SEM)

The samples of *F. vesiculosus* dry biomass were analyzed after and before methyl orange and copper removal using SEM to examine their morphology. The gold-coated dry biomass samples were detected at various magnifications using the accelerating beam voltage of 30 keV.

### Electron dispersive spectroscopy (EDS)

Energy dispersive spectroscopy analysis (EDS) was performed using scanning electron microscope (JEOL, JEM-2100, Japan). EDS was used to determine the contents of elements of *F. vesiculosus* biomass after and before the biosorption process.

## Conclusions

The current study provides an interesting, harmless and environmentally-friendly approach that uses macro-brown alga *Fucus vesiculosus* to remove copper and methyl orange dye simultaneously from aqueous solutions. Box–Behnken design was used to optimize the experimental factors for maximum removal of both MO and copper simultaneously from aqueous solutions using marine alga, *Fucus vesiculosus*, biomass. The maximum removal % was obtained by using 3 g/L of *F. vesiculosus* biomass, MO (60 mg/L), copper (200 mg/L) at pH 7 and incubation time of 60 min with agitation at 200 rpm. *F. vesiculosus* dry biomass can be used as an effective and inexpensive biosorbent for the removal of MO and copper ions from wastewater effluents.
